# Tailoring Structural, Emulsifying, and Interfacial Properties of Rice Bran Protein Through Limited Enzymatic Hydrolysis After High-Hydrostatic-Pressure Pretreatment

**DOI:** 10.3390/foods14020292

**Published:** 2025-01-17

**Authors:** Shirang Wang, Zhen Hua, Tengyu Wang, Guoping Yu, Yu Sun

**Affiliations:** 1 School of Food Engineering, Harbin University, Harbin 150086, China; wangshirang20230076@hrbu.edu.cn; 2 School of Economics and Management, Harbin University, Harbin 150086, China; huazhen@hrbu.edu.cn; 3 College of Food Science, Northeast Agricultural University, Harbin 150030, China; wangtengyu2017@126.com; 4 School of Food Engineering, East University of Heilongjiang, Harbin 150066, China

**Keywords:** rice bran protein, limited enzymatic hydrolysis, emulsifying properties, interfacial properties

## Abstract

We carried out limited enzymatic hydrolysis with trypsin on rice bran protein (RBP) pretreated by high hydrostatic pressure (HHP) in this study. The effects of the degree of hydrolysis (DH) on the structural and emulsifying properties were investigated. The results indicated that the molecular structure of RBP changed after limited enzymatic hydrolysis. The rice bran protein hydrolysate (RBPH, DH8) exhibited a better molecular distribution, a smaller particle size (200.4 nm), a better emulsifying activity index (31.82 m^2^/g), and an improved emulsifying stability index (24.69 min). RBPH emulsions with different DH (0–12) values were prepared. The interfacial properties, such as particle size, the ζ-potential, and the interfacial tension of the emulsions, were measured. Compared to the control, the interfacial properties of the RBPH emulsions were significantly improved after limited enzymatic hydrolysis. The RBPH emulsion at DH8 showed better stability with a smaller emulsion droplet size (2.31 μm), a lower ζ-potential (−25.56 mV), and a lower interfacial tension. This study can provide a theoretical basis for the application of RBP as the plant protein-based emulsifier in the beverage industry.

## 1. Introduction

The emulsifying property of raw material is a crucial factor affecting product quality in the food industry. Emulsification refers to the process of forming a stable emulsion by utilizing emulsifiers to combine two immiscible liquids (e.g., water and oil) [1]. Emulsifiers have extensive applications in the food processing industry, including dairy products, beverages, flavorings, etc. [2]. The taste, texture, and stability of food products can be improved through emulsification, thereby extending their shelf life [3].

With the growing concerns about food health and safety, there is an increasing demand for healthy food ingredients. The rice bran protein (RBP), rich in various essential amino acids and low in allergenicity, is considered a potential high-quality food ingredient with promising applications in the food industry [4,5]. Therefore, the research and development of food ingredients with excellent emulsifying properties is of great significance for the development of the food industry. However, the emulsifying properties of natural RBP are not satisfactory. This is caused by the existence of disulfide bonds, which limits the application of RBP in the food industry [6,7]. To improve the emulsifying properties of RBP, researchers have attempted various modification methods, among which the limited hydrolysis is effective. Limited hydrolysis changes the structural and functional properties of proteins by controlling the hydrolysis conditions, so that the proteins are partially hydrolyzed under the action of specific enzymes. The molecular structure of proteins can be unfolded through limited hydrolysis, exposing more active groups and enhancing their functionality [8,9]. Shuai et al. [10] utilized four proteolytic enzymes to moderately hydrolyze pea protein and investigated the effects of different degrees of hydrolysis (DH) on the structure and function of pea protein. They found that the method of enzymatic modification significantly improved the solubility of pea protein, leading to a significant increase in solubility to 58.14%. Zang et al. [11] found that limited hydrolysis of soybean isolate protein by papain or its mixture with phytase improved the freeze–thaw stability and environmental adaptability. Arteaga et al. [12] revealed that limited enzymatic hydrolysis can alter the molecular weight distribution of pea protein, improving the functional and sensory properties.

High-hydrostatic-pressure (HHP) processing is an emerging technology that can enhance food quality and shelf life; it is seen as a suitable processing method for the food industry. Heat treatment is replaced by pressure processing (100 to 800 MPa); hence, it is called a non-thermal or “cold” pasteurization technique, avoiding the negative effects of heat on the sensory and nutritional attributes of the food products [13]. The protein structure can be modified by HHP processing. The physicochemical forces and interactions between protein molecules of the dispersion systems are affected by HHP, including surface hydrophobicity, electrostatic interactions, disulfide bonds, and hydrogen bonds [14,15].

Currently, population growth and climate change pose huge challenges to agriculture [16]. Hence, green and efficient agricultural product processing is the future development trend in the food industry. There are many studies on individual modifications of plant proteins [17,18,19,20], but there are fewer studies on the combined modification of rice bran protein, especially the combined modification of RBP by HHP and limited enzymatic hydrolysis. In this study, the RBP pretreated by HHP was subjected to limited enzymatic hydrolysis. The influences of DH on the structural and emulsifying properties of rice bran protein hydrolysate (RBPH) were investigated. This was to prepare an emulsifier with good emulsifying properties. A stable RBPH emulsion was fabricated. The interfacial properties of RBPH emulsions with different DH were evaluated. This is meaningful for providing a green and high-value-added processing method for RBP applications.

## 2. Materials and Methods

### 2.1. Materials

Fresh rice bran was obtained from Heilongjiang Great Northern Wilderness Agribusiness Group Co., Ltd. (Harbin, Heilongjiang, China). RBP was laboratory-made. Soybean oil was obtained from Harbin Huikang Food Co., ltd. (Harbin, Heilongjiang, China). Trypsin (3 × 10^4^ U/g) and Nile red were obtained from Sigma-Aldrich Co. (St. Louis, MO, USA). The deionized water was used to prepare solutions. Chemicals and reagents were of analytical grade.

### 2.2. HHP Pretreatment

The HHP equipment (Ren He Electromechanical Engineering Co., Ltd., Shenyang, China) was used for HHP pretreatment at 25 °C and the water was used as a hydrostatic fluid. Then, 200 mL of RBP solution (10 mg/mL) was sealed in a polyethylene plastic bag with the exhaust air. After that, the sample was laid in the HHP equipment for treatment at 200 MPa for 30 min [6].

### 2.3. Preparation of RBPH

For the preparation of RBPH, we referred to Wang et al. [6]. The HHP-pretreated RBP solution (10 mg/mL) was hydrolyzed with trypsin, with pH 8.0, at 37 °C for 0~480 min. The enzyme–substrate ratio was 1:20. The trypsin was inactivated at 90 °C for 10 min. We adjusted the pH of the solution to 7.0 and centrifugated with 8000 rpm for 10 min; the supernatant was lyophilized and stored at −20 °C until use. The lyophilized powder was RBPH [6]. We conducted limited enzymatic hydrolysis of samples for 480 min via the pH-stat method. The DH values of different samples were 0%, 2%, 4%, 8%, and 12% (we show DH0–DH12 in the following). DH values were determined and calculated according to the methods of Zang et al. [11].

### 2.4. Measurement of Hydrolysate Properties

#### 2.4.1. Particle Size Measurement

To measure the effect of limited enzymatic hydrolysis on the particle size of the hydrolysates, the sample was diluted to 10 mg/mL with phosphate-buffered solution (PBS, 10 mmol/L, pH 7.0). The particle size was analyzed by the Mastersizer 2000 (Malvern Instrument Co., Ltd., Worcestershire, UK). The volume average particle size (d_4,3_) was used to characterize the particle size of hydrolysates.

#### 2.4.2. ζ-Potential Measurement

To determine the effect of limited enzymatic hydrolysis on the ζ-potential of the hydrolysates, which helps to assess their surface charge characteristics and stability in solution, the sample was diluted to a concentration of 10 mg/mL using PBS (10 mmol/L, pH 7.0). Then, the ζ-potential was determined with Zetasizer Nano ZS (Malvern Instrument Ltd., Worcestershire, UK).

#### 2.4.3. Sodium Dodecyl Sulfate–Polyacrylamide Gel Electrophoresis (SDS- PAGE) Analysis

SDS-PAGE analysis was used to analyze the effect of limited enzymatic hydrolysis on the primary structure of the hydrolysates. The method was carried out by Wang et al. [6]. For the SDS-PAGE, a 4% stacking gel and a 12% separating gel, containing 0.1% (*w*/*v*) SDS, were used. Electrophoresis was performed at 90 V for the stacking gel and at 120 V for the separating gel. The electrophoretic sample buffer was prepared by mixing 10% glycerol, 0.5 mol/L Tris-HCl buffer (pH 6.8), 5% β-mercaptoethanol, and 1% bromophenol blue. The sample was mixed with the buffer solution and boiled in boiling water for 5 min. It was then cooled to room temperature. After this treatment, 6 μL of each sample was loaded into the gel tank. Following electrophoresis, the gel was stained with Coomassie Brilliant Blue R-250. Once staining was complete, the gel was decolorized using a solution composed of 10% (*v*/*v*) methanol, 10% (*v*/*v*) acetic acid, and 80% distilled water.

#### 2.4.4. Fourier Transform Infrared (FTIR) Spectroscopy Measurements

FTIR methods were used to investigate the effect of limited enzymatic hydrolysis on the secondary structure of the hydrolysates as determined using the FTIR spectrometer (PerkinElmer, Buckinghamshire, UK). The samples were mixed with KBr and pressed into pellets. The scanning range was from 500 to 4000 cm^−1^ and the resolution was 4 cm^−1^ (64 scans). The content of secondary structure was calculated by PeakFit (v4.12) software.

#### 2.4.5. Measurement of the Fluorescence Spectrometry

An F-4500 fluorescence spectrophotometer (Hitachi, Tokyo, Japan) was used to investigate the effect of limited enzymatic hydrolysis on the tertiary structure of the hydrolysates. The sample solution was prepared to the concentration of 10 mg/mL with PBS (10 mmol/L, pH 7.0). The measurement of the fluorescence spectrometry was carried out via excitation at 290 nm according to Liu et al. [21] with an emission wavelength from 300 to 450 nm.

#### 2.4.6. Surface Morphology Analysis

To observe the effect of limited enzymatic hydrolysis on the microstructure of the hydrolysates, the morphology of RBPH was observed by scanning electron microscopy (SEM, Hitachi SU8010, Tokyo, Japan). The samples were sprayed with gold using a sputter coater and observed at an accelerating voltage of 5.0 kV. The final samples were scanned at a magnification of 5.0 k.

#### 2.4.7. High-Performance Size Exclusion Chromatography (HPSEC) Measurements

To analyze the effect of limited enzymatic hydrolysis on the molecular weight distribution of the hydrolysates, the Agilent 1100 liquid chromatograph was used to analyze the molecular weight of RBPH according to the method of Guan et al. [22]. The RBPH solution (10 mg/mL) was filtered by the 0.45 μm cellulose acetate membrane. Then, the sample was injected into the Shodex protein KW-804 column (Shodex Separation and HPLC Group, Tokyo, Japan); the mobile phase was formed by 10 mmol/L PBS (pH 7.0) and 0.3 mol/L NaCl. The flow speed was set at 1 mL/min and the eluent was monitored at 280 nm.

#### 2.4.8. Emulsifying Properties

To measure the emulsifying activity index (EAI) and emulsifying stability index (ESI) of the hydrolysates, assessing their potential as emulsifiers, the procedures of Pearce and Kinsella [23] were used to measure the emulsifying properties of RBPH. The RBPH was diluted to the concentration of 5 mg/mL with PBS (10 mmol/L, pH 7.0). The sample (12 mL) was mixed with soybean oil (4 mL) and homogenized for 1 min at 10,000 rpm to prepare an emulsion. Afterwards, 50 μL of the emulsion was fetched from the bottom place; it was diluted with 5 mL of 0.1% SDS solution and vortexed right away. Finally, the absorbances of the mixture at 0 min and 10 min at 500 nm were measured and recorded. The EAI and ESI were calculated as Equations (1) and (2):

(1) EAIm2g=2×2.303×A0×10010000×0.25×1×0.005

(2) $$\mathrm{ESI}\left(\min\right)=\frac{A_{0}}{A_{0}-A_{10}}\times \left(T_{10}-T_{0}\right)\;$$

where *A*_0_ and *A*_10_ are the absorbance as determined at 0 min and 10 min, respectively. *T*_0_ is 0 min, and *T*_10_ is 10 min.

### 2.5. Preparation of Hydrolysate Emulsion

The emulsion was prepared according to Zhang et al. [24]. The RBPH was dispersed in PBS at a concentration of 10 mmol/L and adjusted to a pH of 7.0. The RBPH solution was stirred for 3 h at room temperature. Subsequently, the RBPH solution was mixed with soybean oil (10%, *v*/*v*) and homogenized at 20,000 rpm for 3 min to prepare a coarse emulsion using a homogenizer (Ultra-Turrax T18, Angni Co., Shanghai, China). This coarse emulsion underwent three cycles of emulsification. These were performed by an ultrasound generator (Scientz-II D, Scientz Biotechnology Co., Ltd., Ningbo, China) operating at 450 W for 3 min per cycle. The ultrasound frequency was set to 20 kHz, with a pulse period of 6 s (4 s on, 2 s off). The final emulsion was then stored in a refrigerator until use.

### 2.6. Measurement of Hydrolysate Emulsion Properties

#### 2.6.1. Measurement of the Droplet Size and ζ-Potential of Emulsion

To measure the droplet size and the ζ-potential of the emulsion, which were critical for evaluating its stability and dispersion, the droplet size and ζ-potential of the emulsion were measured by Wang et al. [25]. The emulsion was diluted 10 times before measurement. Then, the droplet particle size analysis and ζ-potential analysis were performed using Particle Size Analyzer (Microtrac S3500, Microtrac Inc., Krefeld, Germany) and Zetasizer Nano ZS (Malvern Instrument Ltd., Malvern, Worcestershire, UK), respectively.

#### 2.6.2. Turbidity

Turbidity reflected the effect of different DH values on the stability of the RBPH emulsion. The measurement of emulsion turbidity was performed according to the work of Wang et al. [26]. The RBPH emulsion was diluted 100 times by PBS, and the absorbance was recorded at 600 nm via a UV spectrophotometer (TU-1800, Beijing Purkinje General Instrument Co., Ltd., Beijing, China), with the PBS serving as a blank control. The turbidity was calculated as Equation (3):

(3) $$T=\frac{2.302\times A\times V}{I}$$

where *A* is the absorbance of the diluted emulsion at 600 nm, *V* is the dilution factor, and *I* is the optical path (0.01 m).

#### 2.6.3. Contact Angle and Interfacial Tension

The contact angle and interfacial tension were used to reflect the effect of different DH values on the wettability and interfacial behavior of the RBPH emulsion. The methods of contact angle assessment and interfacial tension measurement were taken from the work of Wang et al. [27]. The sitting drop method was utilized to measure the contact angle and interfacial tension of the RBPH emulsion. Generally speaking, the RBPH emulsion was smeared onto a glass slide to form a thin film with a diameter of 10 mm. A drop of water was released at the center of the film using a high-precision syringe, and the image of the droplet was recorded right away, after being dropped from the syringe, using a high-speed camera. The droplet’s profile was numerically solved, and the data were fitted to the Laplace–Young equation to determine the interfacial tension.

#### 2.6.4. Morphology of Emulsion

The morphology of emulsion was used to observe the effect of different DH values on the microstructure of the RBPH emulsion. In this task, an inverted fluorescent microscope (LEICA DMi8, Leica Microsystems, Wetzlar, Germany) was used. The emulsion was stained with Nile red for 30 min in dark conditions to observe its microstructure.

### 2.7. Statistical Analysis

Each experiment was replicated three times, the mean value ± standard deviation was used to express the results obtained using SPSS 22.0 software. Duncan’s test was used to compare the significant differences (*p* < 0.05).

## 3. Results and Discussion

### 3.1. Effect of Limited Enzymatic Hydrolysis on the Physicochemical Properties of RBPH

#### 3.1.1. Relationship Between Hydrolysis Degree and Hydrolysis Time

**Figure 1:**
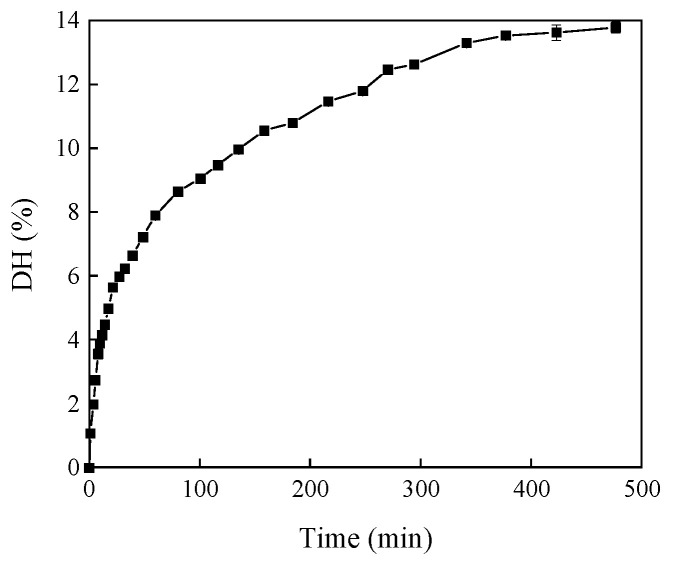
Relationship between DH and hydrolysis time.

The relationship between different the DH levels of HHP-modified RBP and hydrolysis time was studied using the pH stat method. Figure 1 shows the relationship between DH values and the hydrolysis time. During the entire hydrolysis process, as Figure 1 shows, the trypsin hydrolysis becomes intense within 30 min, and the DH changes quickly in the initial hydrolysis stage. At 3.67 min, the DH was 1.99%; at 9.58 min, the DH was 3.9%; and at 27.4 min, the DH reached 5.98%. Subsequently, the change trend of DH gradually slowed down over time, reaching 12.62% at 293.83 min. Afterwards, with the increase in time, the DH curve gradually flattened out. We selected the representative DHs (0, 2%, 4%, 8%, and 12%) for investigation.

#### 3.1.2. Effect of Limited Enzymatic Hydrolysis on the Particle Size of RBPH

**Figure 2:**
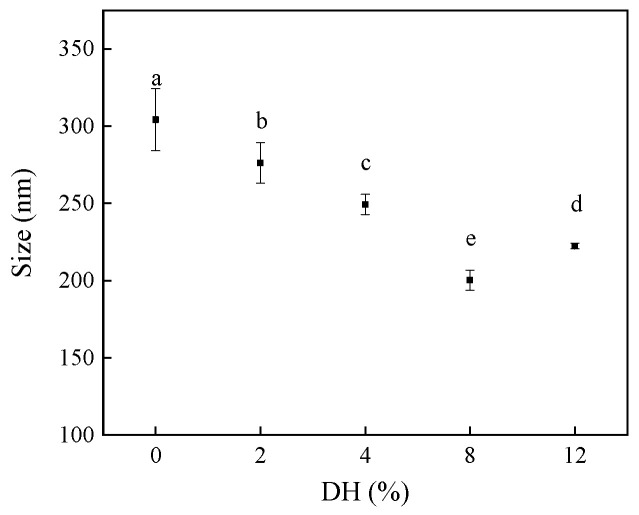
The effect of limited enzymatic hydrolysis on the particle size of RBPH. The error bars indicate the standard deviation obtained from triplicate determinations. Different letters represent significant differences (*p* < 0.05).

The particle size can reflect the microscopic changes in RBPH. Hydrolyzed particles with smaller particle sizes can provide better emulsification performances when they are used as emulsifiers to form the emulsion. Figure 2 shows the changes in the particle size of the RBPH with different DH. values Compared with the solely HHP-modified rice bran protein sample (DH0), the particle sizes of the protein samples hydrolyzed by trypsin showed significant (*p* < 0.05) changes, displaying a trend of first decreasing and then increasing. The particle size of the DH0 was the largest (304.2 nm), while that of the DH8 was the smallest, with a size of 200.4 nm. The research results were consistent with those of Wang et al. [28].

Previous studies show that, with the treatment of HHP, the protein structure can be unfolded, which can expose the protease binding sites for effectively targeted protein hydrolysis [6]. With the increase in DH, the protein molecules were hydrolyzed into smaller protein chains, and the particle size of hydrolysates decreased significantly (*p* < 0.05). The particle size of the DH12 sample (222.4 nm) increased compared to the DH8 sample. This was due to the aggregation of small particles. In addition, the excessive hydrolysis of protein can produce bitter peptides, which can also limit the application of RBPH. Therefore, moderate hydrolysis can provide the RBPH with smaller particle size, which can quickly disperse the RBPH on the interface between continuous and discontinuous phases in solution, thereby improving the functionality of the RBPH.

#### 3.1.3. Effect of Limited Enzymatic Hydrolysis on the ζ-Potential of RBPH

**Figure 3:**
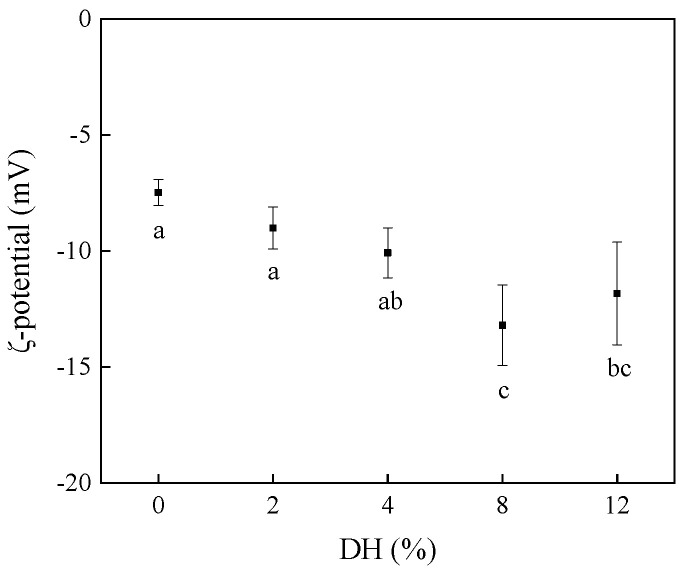
The effect of limited enzymatic hydrolysis on the ζ-potential of RBPH. The error bars indicate the standard deviation obtained from triplicate determinations. Different letters represent significant differences (*p* < 0.05).

ζ-potential is an indicator that shows the charged situation of the particles in solution; the absolute value of the ζ-potential can reflect the stability of an emulsion system. Generally speaking, the greater the absolute value of ζ-potential is, the greater the electrostatic repulsion force of particles in emulsion will be. This can help to resist the aggregation of emulsion droplets caused by van der Waals force and hydrophobic force, thus maintaining the stability of emulsion. The influence of limited enzymatic hydrolysis on the ζ-potential of RBPH is shown in Figure 3. Compared with the DH0, the absolute ζ-potential values of the samples after trypsin hydrolysis showed a trend of first increasing and then decreasing. The absolute ζ-potential value of DH0 sample was the smallest (7.48 mV). As the degree of hydrolysis increased, the absolute ζ-potential value of the RBPH increased gradually, and the ζ-potential value of DH8 sample was the highest (13.20 mV). These results were consistent with the study of Avramenko et al. [29].

The reason the result was that the molecular structure of RBP was unfolded further after trypsin hydrolysis, and the polar groups previously hidden in the protein molecular structure were exposed. Therefore, the surface charge was increased. However, the absolute ζ-potential value of DH12 sample was decreased, which was caused by the aggregation of RBPH particles. This confirmed the reason for the particle size (DH12) increase further. Therefore, moderate hydrolysis can obtain RBPH with high surface charge.

#### 3.1.4. Effect of Limited Enzymatic Hydrolysis on the SDS-PAGE of RBPH

**Figure 4:**
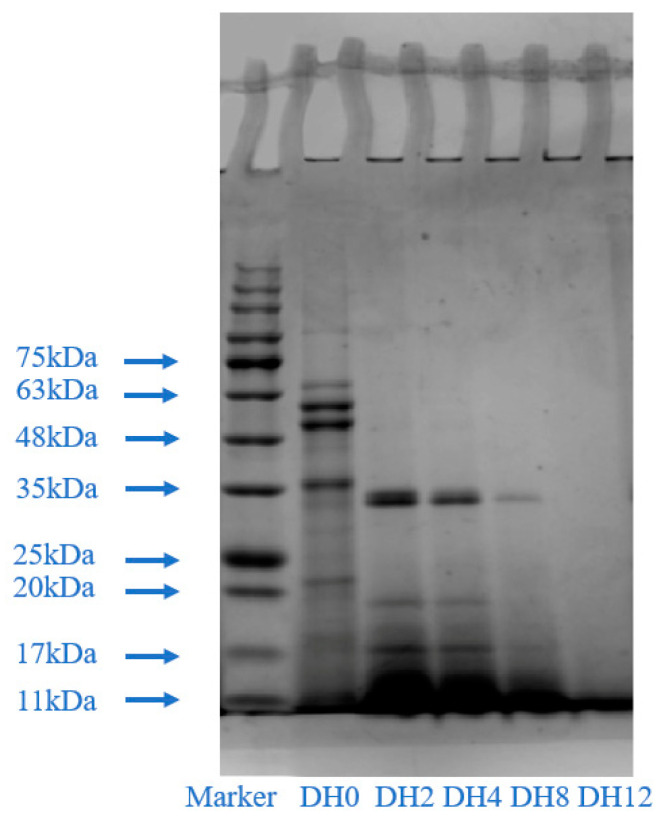
The effect of limited enzymatic hydrolysis on the SDS-PAGE of RBPH.

The SDS-PAGE method was used to investigate the changes in the primary structure of RBPH. As shown in Figure 4, the lane of each sample shows a clear trend of change. The lanes of the DH0 sample existed at bands of 17–20 kDa, 20–25 kDa, 35–48 kDa, separately, and three bands existed between 48 and 75 kDa. The HHP treatment can significantly open the RBP molecular structure at the 17–20 kDa and 20–25 kDa bands. The bands in this study were relatively shallow, which was consistent with the work of Wang et al. [6]. As the degree of trypsin hydrolysis gradually deepened, it was also found that the molecular weight of these two bands gradually decreased until they disappeared. The bands at 35–48 kDa also shifted downwards and then disappeared as the DH increased. The high-molecular-weight bands (48–75 kDa) disappeared at DH2, indicating that trypsin first acted on the protein structure with a higher molecular weight. This also indicated that HHP can unfold the molecular structure of RBP. Then, trypsin hydrolyzed on the cleavage site effectively. The high-molecular-weight bands basically disappeared and protein molecules were distributed below 11 kDa after undergoing thorough hydrolysis (DH12).

The SDS-PAGE can be used to observe the approximate molecular weight distribution of each protein sample with different DH values. After HHP pretreatment, the trypsin can split the protein into smaller proteins by cleaving the peptide bond in the protein. This affects the conformation of higher structures of the protein, enabling the protein to obtain other characteristics or improve its functionalities.

#### 3.1.5. Effect of Limited Enzymatic Hydrolysis on the Secondary Structure of RBPH

**Figure 5:**
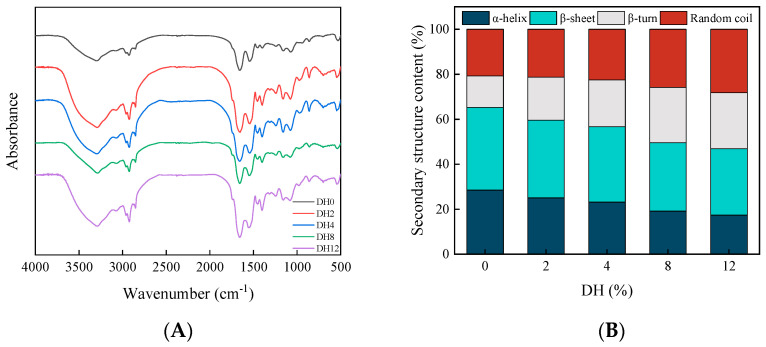
The effect of limited enzymatic hydrolysis on the secondary structure of RBPH. (**A**) FTIR spectra; (**B**) secondary structure content.

The vibrational state changes in protein chemical bonds can be characterized using FTIR spectral information. FTIR spectroscopy was used to detect the changes in the secondary structure at different degrees of hydrolysis. The valuable information on the conformation of protein hydrolysates was reflected within the wavelength range from 500 to 4000 cm^−1^. The FTIR spectra of different DH samples of RBPH are shown in Figure 5A. After limited enzymatic hydrolysis, the characteristic absorption peaks of functional groups of HHP-modified proteins were shifted. The characteristic absorption peak of the O-H tensile vibration of DH0 sample in 3000–3500 cm^−1^ was 3302 cm^−1^. As the degree of hydrolysis increased, this value gradually decreased, and the characteristic peak of DH12 sample underwent blue shift to 3288 cm^−1^. This result indicated that the hydrolysis after HHP pretreatment had an impact on the hydrogen bonds. The samples’ absorption peaks in the range of 2800–3000 cm^−1^ also took the blue shift, decreasing from 2926 to 2922 cm^−1^. The change was related to the stretching motion of C-H, indicating that the trypsin hydrolysis changed the hydrophobic environment of RBP modified by HHP, thereby affecting the structural and functional properties. In addition, the absorption peak of C=O at 1600 to 1700 cm^−1^ of the hydrolysate was also changed. In summary, the trypsin hydrolysis altered the microenvironment of HHP-modified RBP; this led to changes in the secondary structure.

The results of secondary structure content analysis are shown in Figure 5B. Generally speaking, the α-helix and β-sheet are ordered protein secondary structures with high stability, and the β-turn and random coils are disordered structures [30]. Compared to the DH0, the contents of α-helix and β-sheet decreased, whereas the contents of β-turn and random coil increased. The structure of RBP became stretchy and disordered due to the limited enzymatic hydrolysis. Due to this, the RBPH was distributed on the interfacial layer effectively, thus improving the emulsifying properties [31,32]. The structure of RBP changed significantly after limited enzymatic hydrolysis via HHP pretreatment, affecting its physicochemical and functional properties.

#### 3.1.6. Effect of Limited Enzymatic Hydrolysis on the Fluorescence Intensity of RBPH

**Figure 6:**
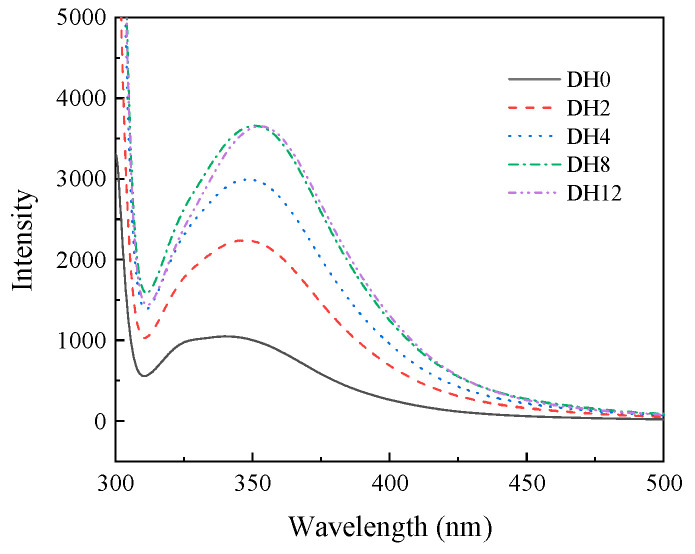
The effect of limited enzymatic hydrolysis on the fluorescence intensity of RBPH.

The changes in the tertiary structure of RBPH samples with different DHs were analyzed by endogenous fluorescence. As shown in Figure 6, the tertiary structure of RBPH changes significantly via trypsin hydrolysis (HHP pretreatment firstly). The λmax of DH0 sample was 340 nm; the λmax of hydrolyzed samples (DH2–DH12) were 347.8 nm, 348.2 nm, 350.6 nm, and 353.0 nm, respectively. The fluorescence intensity of the DH0 sample was 1048.26. Compared to the DH0 sample, the fluorescence intensities of the RBPH sample of DH2–DH12 were significantly increased.

Based on the analysis of experimental data, we concluded that the λmax of the sample hydrolyzed by trypsin was red-shifted. This indicated that when the tryptophan residue of RBPH was exposed to the hydrophilic environment outside, the tertiary structure of the RBPH was changed. Compared to the DH0 sample, the increase in fluorescence intensity after hydrolysis was attributed to the exposure of more aromatic groups to the solvent environment. The fluorescence intensity of the DH12 sample was expected to increase compared to the DH8 sample, but this phenomenon did not appear as expected. This was because, with the increase in DH, the sample hydrolyzed into smaller protein units which aggregated through hydrophobic interactions; then, the exposed aromatic groups cohered into the protein cluster again. It was also possible that hydrolysis increased the flexibility of the protein, which masked the sensitive sites of aromatic amino acids during the process of structural refolding. The trend in changes in the particle size and the ζ-potential can also confirm the reason of this phenomenon in this part.

#### 3.1.7. Effect of Limited Enzymatic Hydrolysis on the Microstructure of RBPH

**Figure 7:**
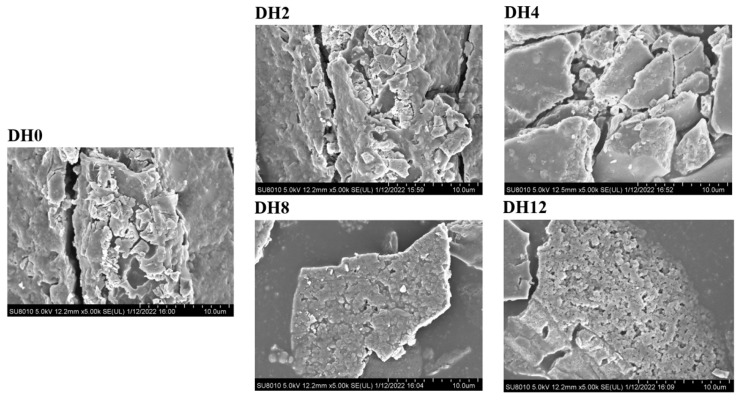
The effect of limited enzymatic hydrolysis on the microstructure of RBPH.

The SEM was used to characterize the microstructural changes in the HHP-modified RBP with different DH values. The results are shown in Figure 7. The DH0 sample exhibited a relatively tight and irregular sheet-like structure. With the increase in the DH values, the morphology of protein flake began to generate some cracks and decompose into small pieces. The particle size became smaller, indicating that RBP was effectively hydrolyzed by trypsin. The micromorphologies of different DH samples were significantly different. It can also be observed from the figure that many small pores are formed on the surface of DH8 and DH12 samples, which may be due to the unfolding in the protein structure caused by trypsin hydrolysis [33]. It can be seen from the SEM image of the DH8 that the surface shape of the protein is smoother and the particle size is more uniform. This can increase the surface area of the hydrolysate and increase the opportunities for interaction between the RBPH and the water. This may have a certain impact on the functional properties of RBPH. Although the particle size of DH12 sample was the smallest, the aggregation of these smaller particles was obvious. This potentially also proves the phenomenon in this study where the particle size of the DH12 was increased, the absolute ζ-potential value was decreased, and the fluorescence intensity was not increased significantly.

#### 3.1.8. Effect of Limited Enzymatic Hydrolysis on the Molecular-Weight Distribution Profiles of RBPH

**Figure 8:**
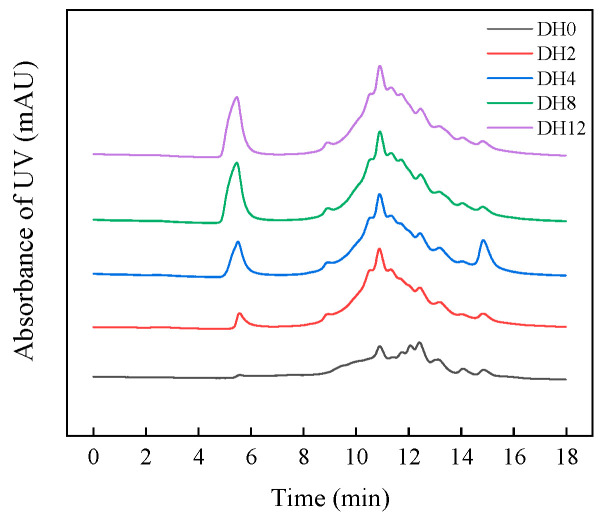
The effect of limited enzymatic hydrolysis on the molecular-weight distribution profiles of RBPH.

The volume exclusion chromatography was used to characterize the molecular weight changes of RBPH with different DH. The molecular weight distribution of DH0–DH12 samples is shown in Figure 8 and Table 1. From Figure 8, it can be seen that with the increase in DH, the peak area around 6 min is gradually increased; around the 11 min, the peak appearance time of each sample is gradually increased. This indicates that enzymatic hydrolysis has an impact on the molecular weight composition of RBPH.

The detailed information on the molecular weight changes of RBPH samples is reflected in Table 1. With the increase in DH, the content of molecular weight between 22 and 33 kDa gradually decreased, while the molecular weight between 18 and 22 kDa increased, indicating that the high-molecular-weight proteins within this range were hydrolyzed into low-molecular-weight proteins. Compared to the DH0 sample, the protein content of DH2–DH12 samples decreased in the region of 16–18 kDa. It was for this reason that the RBPH was hydrolyzed in this region effectively. The molecular weight was obviously not changed in the region less than 14 kDa. The molecular content of each sample gradually was increased with the increase in DH values in the range greater than 33 kDa.

It can be seen that, with the increase in DH values, the RBP modified by HHP was selectively hydrolyzed into lower-molecular-weight hydrolysates. During this process, the proportion of high-molecular-weight protein (22–33 kDa, 16–18 kDa) was gradually decreased, while the content of small molecule hydrolysates was gradually increased. The proportion of protein molecules greater than 33 kDa was increased with the degree of hydrolysis. This meant that the protein aggregation occurred simultaneously during the hydrolysis process. The low-molecular-weight protein hydrolysates were polymerized to macromolecular polymers via disulfide bonds [34]. Our research found that the hydrolysate samples of DH8 and DH12 had better emulsifying properties, with more molecules with molecular weights of 18–22 kDa [6]. It might be that the molecules in this region contributed significantly to improvement in RBPH’s emulsifying properties. Overall, the hydrolysis and the polymerization of protein molecule occurred simultaneously during the process of protein hydrolysis; furthermore, the emulsifying properties of RBPH could be improved by appropriate DH values.

#### 3.1.9. Effect of Limited Enzymatic Hydrolysis on the EAI and ESI of RBPH

**Figure 9:**
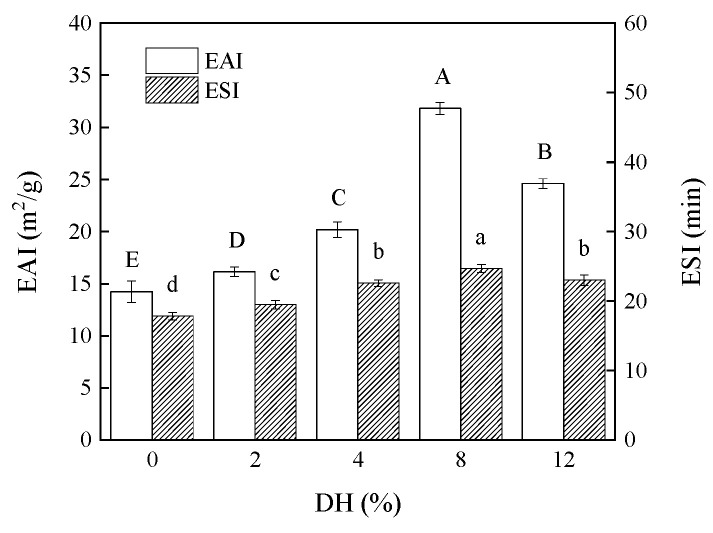
The effect of limited enzymatic hydrolysis on the EAI and ESI values of RBPH. The error bars indicate the standard deviation obtained from triplicate determinations. Different letters represent significant differences (*p* < 0.05).

The purpose of this study was to screen the RBPH sample with better emulsifying properties as an emulsifier. Therefore, the changes in the emulsifying properties of RBPH with different DH values were investigated. The EAI and ESI values of different DH samples are exhibited in Figure 9. The EAI and ESI values of the RBPH samples were significantly increased with the increase in DH (*p* < 0.05) compared to DH0, but showed a slight downward trend at DH12.

There were two main reasons for this phenomenon: (1) With the increase in DH values, the protein molecules were hydrolyzed into smaller particles, the flexibility was increased, and the interfacial tension was reduced. The protein molecules can quickly form an interfacial layer on the O/W interface. (2) The structure of RBP was initially unfolded by the HHP modification, and then the hydrophobic groups were exposed. At this stage, the hydrophobicity was positively correlated with the emulsification of the protein [24]. Nevertheless, excessive hydrolysis had a negative effect on the emulsification of RBPH. Specifically, the molecules of RBPH with excessively low molecular weights were unfolded incompletely and were difficult to position on the O/W interface, resulting in a thin and unstable interface film. Furthermore, excessive hydrolysis reduced the electrostatic repulsion that normally maintained the stability of the interface, which in turn led to a decline in emulsification [35].

### 3.2. Effect of Limited Enzymatic Hydrolysis on the Interfacial Properties of RBPH Emulsion

#### 3.2.1. Effect of Limited Enzymatic Hydrolysis on the Droplet Size of RBPH Emulsion

**Figure 10:**
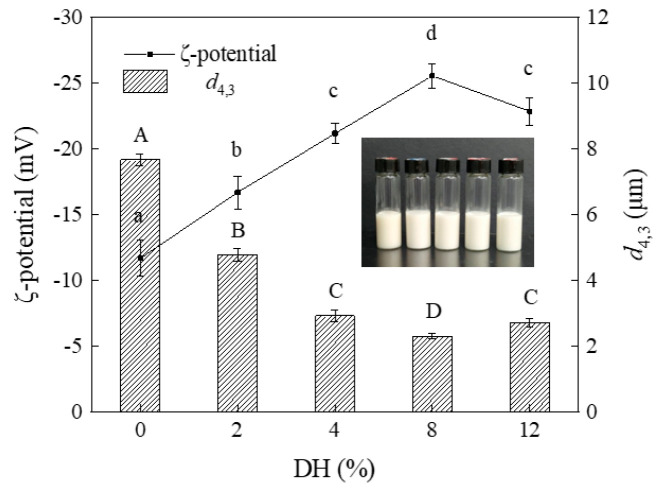
The effect of limited enzymatic hydrolysis on the droplet size and ζ-potential of the RBPH emulsion. The error bars indicate the standard deviation obtained from triplicate determinations. Different letters represent significant differences (*p* < 0.05).

The size of emulsion droplet exerts a significant influence on the emulsion system’s stability. In Figure 10, with the increase in DH (0–12), the emulsion droplet size shows an obvious decreasing trend, and the average droplet sizes of the emulsions decrease significantly (*p* < 0.05) (from 7.67 μm to 2.31 μm). The above results indicated that the RBPH emulsions promoted the formation of smaller emulsion droplets compared with the RBP emulsion without the hydrolysis treatment. Moderate hydrolysis after HHP pretreatment led to better unfolding of the protein structure and smaller droplets of RBPH emulsion. This was because the limited enzymatic hydrolysis can improve the interfacial properties of RBP and increase the migration rate of RBPH on the O/W interface, which was conducive to the formation of smaller-sized emulsion droplets [36]. The reduction in the average droplet size is usually favorable in terms of improving the stability of the emulsion. Smaller droplets imply a larger specific surface area. This can facilitate the enhancement of inter-particle interactions and thus keep the emulsion stable. The limited enzymatic hydrolysis after HHP pretreatment can produce smaller RBPH. This was useful for forcing the RBPH to spread on the interface efficiently [37].

However, the emulsion droplet size increased to 2.73 μm at DH12. Excessive hydrolysis (DH12) caused the RBPH to lose the structural optimum and affected its emulsifying properties [38]. This saw the originally exposed hydrophobic groups re-encapsulated in the spatial structure of the protein. The densification and integrity of the interfacial film were affected due to the increased aggregation of interfacial proteins [39]. This led to the aggregation and the flocculation of emulsion droplets; then, the emulsion droplets became larger. The results of this study were consistent with those of Liu et al. [40].

#### 3.2.2. Effect of Limited Enzymatic Hydrolysis on the ζ-Potential of RBPH Emulsion

The ζ-potential can reflect an emulsion’s stability. The electrostatic repulsion between droplets plays a pivotal role in maintaining the stability of emulsion. The absolute value of ζ-potential reflects the net charge amount on the surface of the droplets, thereby quantifying the strength of electrostatic interactions. Therefore, this serves as an indicator with which to evaluate the emulsion system’s stability.

The experimental findings (Figure 10) demonstrated that as the DH value increased, the absolute value of ζ-potential of RBPH emulsion exhibited the trend of first increasing and then decreasing significantly (*p* < 0.05). The maximum absolute value of ζ-potential was 25.56 mV at DH8. A reduction in the absolute value of ζ-potential appeared at DH12 (22.86 mV). For the emulsion system, the decrease in net charge implies weakened interactions between oil droplets [41]. Generally, a higher absolute value of ζ-potential signifies stronger electrostatic repulsion between particles in the emulsion, which is conducive to maintaining the emulsion’s stability [42]. However, excessive enzymatic hydrolysis may induce the flocculation and the aggregation between emulsion droplets, negatively affecting the potential of the emulsion. This thus affects stability.

#### 3.2.3. Effect of Limited Enzymatic Hydrolysis on the Turbidity of RBPH Emulsion

**Figure 11:**
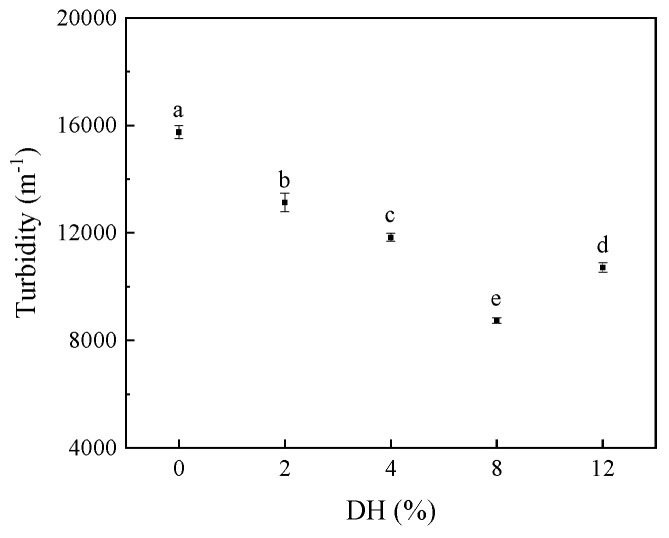
The effect of limited enzymatic hydrolysis on the turbidity of RBPH emulsion. The error bars indicate the standard deviation obtained from triplicate determinations. Different letters represent significant differences (*p* < 0.05).

The turbidities of RBPH emulsions prepared with different DH values are illustrated in Figure 11. As DH values increased, the turbidity of RBPH emulsion exhibited a trend of initially decreasing and subsequently increasing. Compared to the RBP emulsion without enzymatic hydrolysis, all the RBPH emulsions showed significant (*p* < 0.05) reductions in turbidity. The emulsion exhibited the lowest turbidity at DH8.

However, excessive enzymatic hydrolysis may also have a negative effect on the emulsion’s stability. The turbidity increased at DH12. This was because the excessive enzymatic hydrolysis treatment potentially led to the aggregation of interfacial proteins and the disruption of the O/W interface. It made the emulsion more susceptible to stratification or sedimentation, leading to increased turbidity. In general, turbidity is correlated with the droplet size and the droplet aggregation status in emulsions [43]. Typically, a stable emulsion exhibits lower turbidity [44]. The results of turbidity were consistent with the findings of droplet size and ζ-potential in this study.

#### 3.2.4. Effect of Limited Enzymatic Hydrolysis on Contact Angle and Interfacial Tension of RBPH Emulsion

**Figure 12:**
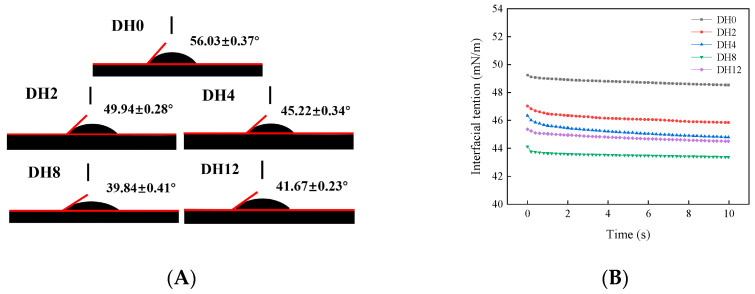
The effect of limited enzymatic hydrolysis on the contact angle (**A**) and interfacial tension (**B**) of RBPH emulsions.

The contact angle can be used to access the hydrophilicity and hydrophobicity of emulsion droplets. Through the experimental results (Figure 12A), it is shown that the contact angle of RBPH emulsion (DH2–12) changes obviously. The contact angle of the DH0 emulsion was relatively large (56.03°), indicating the poor wettability of the emulsion interface. The contact angles of the DH2–12 emulsions exhibited a decreasing trend with increasing DH values. Specifically, the contact angle of the emulsion sample reached the minimum value (39.84°) at DH8, demonstrating better wettability. Moderate hydrolysis enhanced the wettability of emulsion, thereby enhancing stability. This was attributed to the fact that limited enzymatic hydrolysis after HHP can significantly improve the rapid adsorption and arrangement of emulsifier molecules on the oil–water interface, thus improved the stability of the emulsion. Nevertheless, when the hydrolysis was excessive (DH12), the contact angle increased inversely. The arrangement of emulsifier molecules on the O/W interface became disordered. This was caused by the excessive hydrolysis, ultimately reducing the stability of the emulsion.

Interfacial tension is one of the crucial parameters of emulsion. It significantly impacts the emulsion’s formation, dispersion, and interactions with other substances. Specifically, the emulsion with lower interfacial tension tends to form a more stable emulsion. This is important for preventing the emulsion from undergoing phase separation and precipitation effectively. The interfacial tensions of RBPH emulsion are depicted in Figure 12B. As the experimental results indicated, the interfacial tension initially decreased and subsequently increased as the DH increased. Notably, the interfacial tension was the minimum at DH8, indicating that this was the most stable emulsion of all. The appropriate hydrolysis treatment can effectively reduce the interfacial tension and improve the stability of the emulsion. This result can be attributed to the fact that appropriate hydrolysis facilitates the diffusion and adsorption of emulsifier molecules on the O/W interface, forming a more stable and thicker interfacial layer. The appropriate hydrolysis improved the molecular arrangement on the emulsion surface, rendering it more uniform [45]. This resulted in a more organized arrangement of molecules on the surface of the emulsion and a weakening of the intermolecular interaction forces, which reduced the surface tension. However, when the hydrolysis was excessive, the interfacial tension increased. This was because excessive hydrolysis affected the dispersion of RBPH on interfacial layer. It can be concluded that the moderate enzymatic hydrolysis after HHP has a positive effect on optimizing emulsion stability.

#### 3.2.5. Effect of Limited Enzymatic Hydrolysis on the Microscopic Morphology of RBPH Emulsion

**Figure 13:**
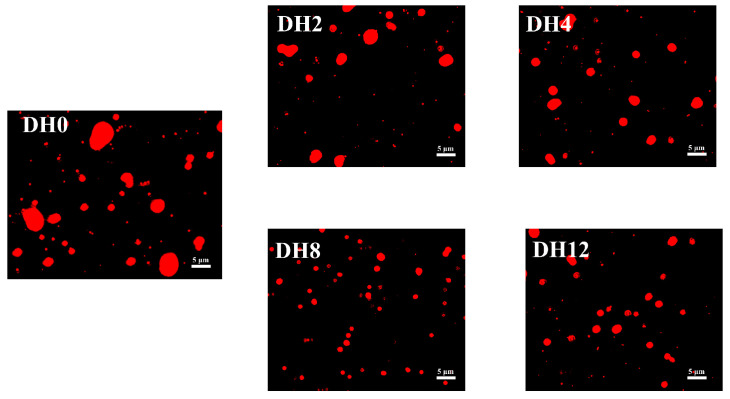
The effect of limited enzymatic hydrolysis on the microscopic morphology of RBPH emulsion.

The microscopic morphology of the emulsion was observed via an inverted fluorescent microscope. The microstructure was significantly affected by limited enzymatic hydrolysis. Figure 13 shows that the emulsion particle size can be refined effectively by limited enzymatic hydrolysis. The microstructure was improved, which was conducive to improving emulsion stability. At DH8, the emulsion particle size was the smallest and was uniformly distributed. This observation was consistent with our findings regarding the average droplet size.

## 4. Conclusions

In this study, the RBP pretreated with HHP was subjected to limited enzymatic hydrolysis by trypsin. An RBPH (DH8) was prepared with better emulsifying properties. The RBPH (DH8) was used as an emulsifier to construct a stable emulsion system. The interfacial stabilization mechanisms of the emulsions prepared by RBPH were elucidated. The experimental results demonstrated that the limited enzymatic hydrolysis altered the structural and molecular characteristics of RBP. The molecular structure of the RBP was unfolded, exposing more hydrophilic groups. This enhanced their dispersibility and interfacial activity in aqueous phases, leading to a marked improvement in emulsification properties. The limited enzymatic hydrolysis after HHP effectively reduced the interfacial tension, promoting the uniform dispersion and stabilization of oil droplets. The emulsion’s droplet size, ζ-potential, and interfacial tension results all indicated that the prepared emulsions had better stability. In conclusion, the RBPH prepared in this study can be used as a novel plant-based emulsifier in the beverage industry, and the RBPH emulsion has potential in the research of nutrient delivery. Moving forward, we will continue to focus on the study of RBPH as an emulsifier with implications for the processing stability and storage stability of emulsion.

## Figures and Tables

**Table 1:** The effect of limited enzymatic hydrolysis on the molecular-weight distribution of RBPH. The different letters represent significant differences (*p* < 0.05).

Samples	Percentage Area of Peak (%) Corresponding to Retention Time/Molecular-Weight Distribution (min, kDa)				
	<10 min	10–11 min	11–12 min	12–13 min	>13 min
	>33 kDa	22–33 kDa	18–22 kDa	16–18 kDa	<14 kDa
DH0	0.75 ± 0.01 ^e^	41.05 ± 0.10 ^a^	13.59 ± 0.02 ^d^	25.85 ± 0.11 ^a^	18.76 ± 0.12 ^b^
DH2	6.51 ± 0.03 ^d^	40.31 ± 0.05 ^b^	22.74 ± 0.06 ^c^	11.69 ± 0.08 ^b^	18.75 ± 0.07 ^b^
DH4	9.89 ± 0.07 ^c^	36.41 ± 0.13 ^c^	22.72 ± 0.05 ^c^	9.92 ± 0.04 ^d^	21.06 ± 0.05 ^a^
DH8	16.16 ± 0.11 ^a^	33.69 ± 0.07 ^d^	23.68 ± 0.09 ^b^	10.18 ± 0.08 ^c^	16.29 ± 0.09 ^d^
DH12	13.26 ± 0.06 ^b^	30.12 ± 0.02 ^e^	27.04 ± 0.05 ^a^	11.71 ± 0.03 ^b^	17.87 ± 0.02 ^c^

## Data Availability

The original contributions presented in this study are included in the article. Further inquiries can be directed to the corresponding authors.
